# Association between meteorological factors and blood pressure variability in northern Guizhou, China

**DOI:** 10.3389/fpubh.2026.1888902

**Published:** 2026-08-07

**Authors:** Yiyue Tang, Yunzhen Lei, Xingli Hu, Yukun Zhang, Yi Yao, Xinyue Chen, Xiaohua An, Qingxian Tu, Qianfeng Jiang

**Affiliations:** 1 Department of Cardiovascular Medicine, The Third Affiliated Hospital of Zunyi Medical University (The First People's Hospital of Zunyi), Zunyi, Guizhou Province, China; 2 Zunyi Medical University, Zunyi, Guizhou Province, China; 3 Department of Cardiovascular Medicine, Guizhou Aerospace Hospital (Affiliated Aerospace Hospital of Zunyi Medical University), Zunyi, Guizhou Province, China

**Keywords:** blood pressure variability, distributed lag non-linear model, DLNM, hypertension, meteorological factors

## Abstract

Hypertension is a major risk factor for cardiovascular mortality worldwide, and its pathogenesis is closely related to environmental exposures. However, most previous studies have focused on the effects of air pollution on resting blood pressure. This study investigated the associations between short-term exposure to meteorological factors and blood pressure variability (BPV). Ambulatory blood pressure monitoring data were obtained from 4,355 patients, and indices of systolic and diastolic BPV were calculated. Daily meteorological variables included relative humidity, shortwave radiation, and temperature. Distributed lag non-linear models (DLNMs) were used to estimate the associations between meteorological exposures and BPV. Over a maximum lag of 14 days, higher relative humidity and lower shortwave solar radiation were positively associated with greater BPV. Exploratory subgroup analyses yielded larger estimates in some daily series stratified by age, sex, smoking status, and alcohol consumption; however, these findings were based on aggregated data and involved multiple comparisons. In this hospital-based population, city-level relative humidity and shortwave radiation were associated with daily mean BPV. These findings should be interpreted as ecological associations rather than evidence of causal effects or confirmed individual susceptibility. Validation in multicenter studies incorporating individual-level exposure assessment is warranted.

## 1 Introduction

Increasing evidence has highlighted the close relationship between the living environment and human health. Evidence-based studies have shown that environmental exposures, including air pollution, temperature, and humidity, exert substantial epidemiological effects on human health (1, 2). The World Health Organization estimates that approximately 9 million deaths each year are attributable to unhealthy environmental conditions (3). Exposure to environmental stressors is increasingly recognized as a secondary contributor to cardiovascular diseases, including hypertension (4).

Hypertension is one of the most prevalent chronic noncommunicable diseases worldwide and represents a major public health threat. According to the World Health Organization, more than 1.2 billion people worldwide have hypertension, with an increasing prevalence among younger individuals and a continuing upward trend. The number of affected individuals is projected to reach 1.5–1.7 billion by 2030 (5). As a major risk factor for cardiovascular disease, hypertension has a complex and multifactorial pathophysiology involving dysregulation of neural control, vascular dysfunction, impaired renal regulation, inflammation, and oxidative stress (6–8).

Numerous studies have linked meteorological factors to elevated systolic and diastolic blood pressure. In a Chinese study of 9,272 participants, compared with the exposure combination associated with the lowest blood pressure percentile, exposure to 4.6 °C and 57.9% relative humidity was associated with increases of 12.05 mmHg [95% confidence interval (CI), 11.63–12.42] in systolic blood pressure and 6.67 mmHg (95% CI, 6.43–6.88) in diastolic blood pressure. Exposure to 22.5 °C and 79.2% relative humidity was associated with corresponding increases of 11.75 mmHg (95% CI, 10.32–13.08) and 6.47 mmHg (95% CI, 5.65–7.25), respectively (9). A short-term prospective study identified both extremely high and extremely low temperatures as risk factors for mortality among patients with hypertension, with effect estimates of 1.144 and 1.122, respectively. Stratified analyses indicated that older adults and women were more susceptible to extremely low temperatures, whereas extremely high relative humidity had a more pronounced protective association in both groups (10). A Swiss study investigating solar radiation and blood pressure reported that women with low and moderate sun exposure had 41 and 15% higher odds of hypertension, respectively, than women with the highest sun exposure (11). Previous research has also shown that BPV, a key measure of blood pressure fluctuations, is closely associated with an increased risk of cardiovascular disease (12). Among patients receiving routine antihypertensive treatment, greater systolic BPV is associated with a higher risk of cardiovascular events (13). BPV is also an important risk factor for target-organ damage and adverse clinical outcomes in patients with hypertension (14–16). Current clinical management primarily focuses on controlling peak blood pressure, whereas the prevention of abnormal blood pressure fluctuations remains insufficiently addressed.

Blood pressure variability (BPV) refers to the magnitude and pattern of blood pressure fluctuations over a defined period and reflects the dynamic capacity of the cardiovascular system to respond to internal and external stimuli (17). It is commonly quantified using the coefficient of variation. According to the monitoring interval, BPV can be classified as very short-term variability (beat-to-beat fluctuations), short-term variability (fluctuations within 24 h), and long-term variability (fluctuations over months or during long-term follow-up) (18). BPV is regulated by the autonomic nervous system and the renin-angiotensin-aldosterone system (RAAS) (19). Hypertension-related organ damage is a major determinant of adverse cardiovascular events and all-cause mortality (20). In clinical practice, target-organ damage may continue to progress in some patients despite adequate control of mean blood pressure, underscoring the independent pathological importance of BPV. A study of 6,632 participants suggested that arterial stiffness may precede increases in systolic BPV and that their combined effects predict incident cardiovascular disease. Participants with persistently high systolic BPV had 28–46% higher risks of cardiovascular disease, coronary heart disease, stroke, atrial fibrillation, heart failure, chronic kidney disease, and all-cause mortality (21). Moreover, home BPV measured during colder seasons showed a stronger association with subsequent cardiovascular disease incidence than BPV measured during other seasons (22). Accumulating evidence indicates that BPV is not only an independent predictor of target-organ damage but may also accelerate its development by aggravating the vicious cycle between hypertension and arterial stiffness (23, 24). BPV is influenced by multiple factors, including emotional state, age, sex, smoking, and the use of certain antihypertensive medications (25). An observational study of 172 participants found that morning indoor temperature fluctuations exceeding 1.1 °C significantly increased BPV (26). A retrospective cross-sectional study also reported a higher risk of increased BPV at higher levels of air pollution exposure (27).

BPV is not a static parameter; rather, it fluctuates continuously over time as a result of interactions among environmental exposures, behavioral factors, and intrinsic cardiovascular regulatory mechanisms (28). Previous studies have largely focused on the associations between environmental factors and absolute blood pressure levels. Relatively few studies have examined environmental determinants of blood pressure fluctuations, particularly the effects of meteorological factors on 24-h BPV in large populations.

Previous environmental epidemiological studies have predominantly evaluated mean blood pressure rather than 24-h ambulatory BPV. In addition, most studies have assessed a single exposure and have rarely characterized both non-linear exposure-response relationships and multiday lag structures. Evidence regarding the associations of relative humidity and shortwave radiation with BPV is particularly limited, as is region-specific evidence. Zunyi, Guizhou Province, has a humid subtropical monsoon climate characterized by high relative humidity and low shortwave radiation (29). Therefore, using the ambulatory blood pressure database of Guizhou Aerospace Hospital and a DLNM framework, this study investigated the associations between meteorological factors and 24-h BPV among patients with primary hypertension in Zunyi. We further evaluated the lagged and cumulative associations of meteorological exposures with BPV. The findings may provide evidence to support blood pressure management and weather-related health interventions for patients with hypertension in this region and may help reduce the risk of cardiovascular complications associated with excessive BPV.

## 2 Materials and methods

### 2.1 Study area

Zunyi is located in northern Guizhou Province, in the transitional zone between the Yunnan-Guizhou Plateau and the Sichuan Basin, at approximately 27.70°N and 106.93°E. The terrain varies substantially in elevation, with a mean altitude of approximately 900 m, and the permanent resident population is approximately 6.5 million. The region has a humid subtropical monsoon climate, with an annual mean temperature of 12.6–18.0 °C, annual precipitation of 1,000–1,300 mm, and 1,000–1,300 h of sunshine per year. It is characterized by abundant rainfall, high humidity, relatively low solar shortwave radiation, and pronounced vertical climatic variation. The annual mean relative humidity in Zunyi is 78–80%, markedly higher than the national average of 68–70%, placing the region among the high-humidity climatic zones of China. Annual mean shortwave radiation (total solar radiation) is approximately 3,250–3,500 MJ/m^2^, corresponding to only 70–78% of the national average and representing one of the low-solar-radiation regions in China.

### 2.2 Data sources

The ambulatory blood pressure database of Guizhou Aerospace Hospital was searched continuously for the period from 2023 to 2025. A total of 4,355 patients diagnosed with primary hypertension were included. The following BPV indices were extracted: 24-h diastolic blood pressure coefficient of variation (24HDBPCV), 24-h systolic blood pressure coefficient of variation (24HSBPCV), daytime diastolic blood pressure coefficient of variation (DAYDBPCV), daytime systolic blood pressure coefficient of variation (DAYSBPCV), nighttime diastolic blood pressure coefficient of variation (NDBPCV), and nighttime systolic blood pressure coefficient of variation (NSBPCV). Daily meteorological data were obtained from the China Meteorological Administration (30) and included daily mean temperature (°C), relative humidity (%), and shortwave radiation (MJ/m^2^). Daily mean concentrations of ambient air pollutants were obtained from the National Urban Air Quality Real-time Publishing Platform (31). The pollutants assessed were PM2.5 (μg/m^3^), PM10 (μg/m^3^), SO_2_ (μg/m^3^), NO_2_ (μg/m^3^), CO (mg/m^3^), and the maximum 8-h average ozone concentration (O_3_-8h; μg/m^3^).

### 2.3 Study population and data linkage

This study included ambulatory blood pressure monitoring records from patients with hypertension who underwent monitoring at Guizhou Aerospace Hospital between 2023 and 2025. The study was approved by the institutional ethics committee (approval no. {2024}KY-53).

The inclusion criteria were as follows: (1) age >18 years; (2) a diagnosis of primary hypertension according to the Chinese Guidelines for the Prevention and Treatment of Hypertension (2024 edition); and (3) long-term residence in the central urban area of Zunyi. The exclusion criteria were as follows: (1) hypertensive crisis or secondary hypertension; (2) an acute illness or hemodynamic instability, including acute myocardial infarction, acute decompensated heart failure, or acute coronary syndrome; (3) severe chronic disease, including acute or chronic hepatic insufficiency, malignant tumors, or hyperthyroidism; (4) autoimmune disease; and (5) incomplete 24-h ambulatory blood pressure monitoring.

Blood pressure was measured using a calibrated, fully automated ambulatory blood pressure monitor (Yuwell YE680A). Trained staff placed the cuff on the upper left arm over the brachial artery. The monitoring period was divided into daytime and nighttime intervals. Blood pressure was measured every 30 min from 08:00 to 22:00 and every 60 min from 22:00 to 08:00. To ensure data reliability, at least 32 valid measurements were required during the 24-h monitoring period. A valid recording required an overall success rate of at least 70%, with at least 20 valid daytime measurements and at least 7 valid nighttime measurements; otherwise, monitoring was repeated. Participants were permitted to continue their usual daily activities but were instructed to avoid vigorous exercise during monitoring. Mean systolic and diastolic blood pressure values and the corresponding coefficients of variation were calculated for the 24-h, daytime, and nighttime periods. Hypertension based on ambulatory monitoring was defined as a 24-h mean systolic blood pressure ≥130 mmHg and/or diastolic blood pressure ≥80 mmHg, a daytime mean systolic blood pressure ≥135 mmHg and/or diastolic blood pressure ≥85 mmHg, or a nighttime mean systolic blood pressure ≥120 mmHg and/or diastolic blood pressure ≥70 mmHg.

The date on which ambulatory blood pressure monitoring commenced was defined as the index date. Individual BPV indices from records initiated on the same date were averaged to obtain daily mean values. Each record was then linked to city-level meteorological and air pollution data for the index date and the preceding 1–14 days. Thus, participants monitored on the same day shared the same exposure history. The final analysis was therefore explicitly defined as a daily ecological time-series analysis.

### 2.4 Statistical analysis

#### 2.4.1 Descriptive analysis

Air pollutant concentrations and meteorological variables during the study period were summarized using minimum, maximum, and mean values. Blood pressure and BPV indices are presented as the mean ± standard deviation (SD), whereas categorical baseline characteristics are reported as counts and percentages.

#### 2.4.2 Spearman correlation analysis

Spearman correlation analysis was used to assess correlations between air pollutants and meteorological variables. A correlation coefficient >0.70 was considered indicative of a strong correlation. Highly correlated variables were excluded from model fitting as appropriate to reduce potential multicollinearity (32).

#### 2.4.3 Statistical model

DLNMs were used to characterize the potentially complex associations between meteorological factors and BPV among patients with primary hypertension. Previous studies have shown that the health effects of environmental exposures are not necessarily restricted to the day of exposure; rather, they may emerge gradually over several subsequent days or longer. Accordingly, DLNMs were fitted to evaluate the associations of temperature, relative humidity, and shortwave radiation with BPV (33).

A key feature of the DLNM is the cross-basis function, which simultaneously represents the exposure and lag dimensions (33). The lag dimension describes the time interval between exposure and the occurrence of a health outcome and therefore captures temporal changes in the estimated effect. The exposure dimension represents the gradient of exposure levels at a given time and enables quantification of differences in the health association across exposure intensities. After the cross-basis matrix was constructed and incorporated into the model, predictions and effect estimates were obtained using the crosspred function.

Natural spline functions with selected degrees of freedom (df) were used to control for potential confounders, including PM2.5 and PM10. Covariates were selected according to the results of the Spearman correlation analysis. Following previous research (34), the df for meteorological variables, confounders, and long-term time trends were selected by minimizing the Akaike information criterion (AIC). Indicator variables for public holidays and day of the week were also included to control for their potential effects on BPV. The basic model for each meteorological exposure was specified as follows:


$$\begin{array}{l} \mathrm{MYt}=\alpha +\mathrm{\beta Rt},l+\mathrm{ns}\;(\text{timet},{\mathrm{df}}^{*}\;\text{years})+\sum \mathrm{ns}(\mathrm{Xi},\mathrm{df})+\\ \kappa \text{holidayt}+\eta \text{dowt} \end{array}$$

In this model, t denotes day t in the study time series; MYt is the mean BPV coefficient in the study population on day t; α is the model intercept; Rt,l is the two-dimensional cross-basis function constructed within the DLNM framework to simultaneously model the non-linear exposure-response relationship and lagged association between the meteorological factor and BPV; and l is the maximum lag, which was set at 14 days on the basis of previous studies (35). The term ns(timet, df × years) controls for the long-term temporal trend, where df is the number of degrees of freedom per year and years is the total study duration. The term Σns(Xi, df) represents natural cubic spline functions used to control for air pollutants such as PM10, PM2.5, and NO_2_. The terms holidayt and dowt represent fixed effects for public holidays and day of the week, respectively.

#### 2.4.4 Subgroup analysis

Analyses were stratified by sex (male or female), age (<60 or ≥60 years), alcohol consumption (yes or no), and smoking status (yes or no). Differences between subgroups were evaluated using a Z test (36), with statistical significance assessed using the following formula:


$$Z=(Q1-Q2)/\sqrt{SE_{2}^{1}+SE_{2}^{2}}$$

#### 2.4.5 Sensitivity analysis

To assess the robustness of the findings, sensitivity analyses were conducted by increasing the df for meteorological variables from 3 to 5 and the df for the temporal trend from 7 to 9.

The median level of each meteorological factor was used as the reference (37) when estimating changes in BPV-related indices. In addition, effect estimates were calculated for different lag days and exposure percentiles (P1 and P99) to systematically assess lag-specific and cumulative lagged associations between meteorological factors and BPV. All statistical analyses and model fitting were performed in R version 4.4.2, and a two-sided *p* value <0.05 was considered statistically significant.

## 3 Results

### 3.1 Meteorological characteristics of Zunyi and characteristics of the study population

A total of 4,355 participants were included, comprising 2,224 women (51.07%) and 2,131 men (48.93%). Among them, 2,462 participants (56.53%) were aged <60 years and 1,893 (43.47%) were aged ≥60 years; 1,641 (37.68%) were smokers and 2,714 (62.32%) were nonsmokers; and 1,205 (27.67%) reported alcohol consumption whereas 3,150 (72.33%) did not. General baseline characteristics are presented in Table 1, and ambulatory blood pressure and BPV indices are shown in Table 2.

**Table 1:** Baseline characteristics of the study population.

Variable	Mean ± SD or n (%)
Age, years, mean ± SD	58.23 ± 13.44
Male, *n* (%)	2,131 (48.93%)
Female, *n* (%)	2,224 (51.07%)
Age <60 years, *n* (%)	2,462 (56.53%)
Age ≥60 years, *n* (%)	1,893 (43.47%)
Current or former smoking, *n* (%)	1,641 (37.68%)
Alcohol consumption, *n* (%)	1,205 (27.67%)

**Table 2:** Ambulatory blood pressure indices of the study population (mean ± SD).

Variable	Mean (mmHg)	SD	BPCV (%)
24HSBP	137.68	17.90	13 ± 3
24HDBP	86.4	14.69	17 ± 5
DAYSBP	138.22	17.97	13 ± 4
DAYDBP	88.13	14.10	16 ± 5
NSBP	135.47	14.90	11 ± 4
NDBP	82.19	11.51	14 ± 5

**Figure 1:**
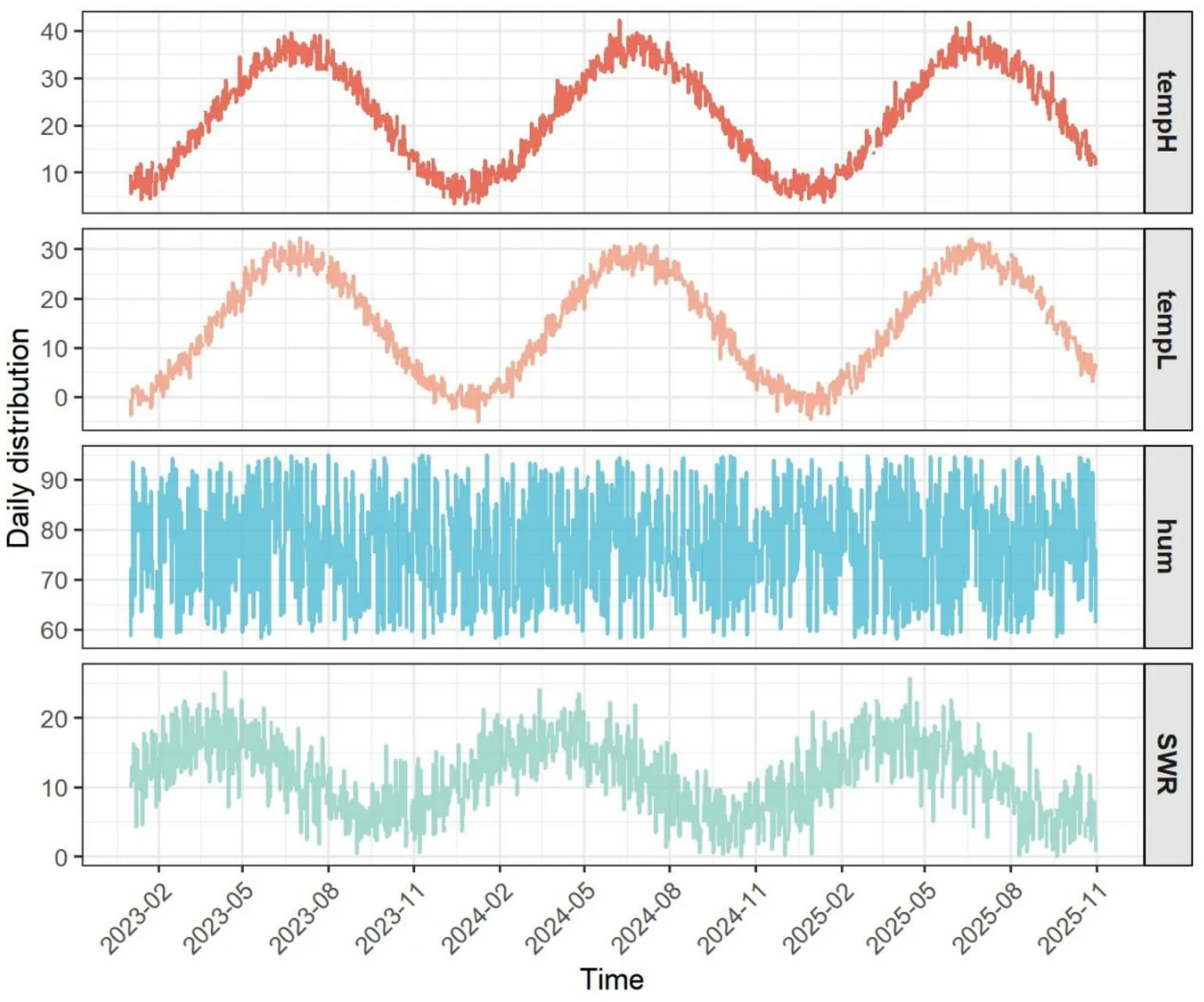
Daily time series of relative humidity, solar shortwave radiation, daily minimum temperature, and daily maximum temperature in Zunyi from 2023 to 2025.

From 2023 to 2025, the mean relative humidity (Hum) in Zunyi was 77.14%, the mean shortwave radiation (SWR) was 11.46 MJ/m^2^, the mean daily low temperature (TempL) was 14.02 °C, and the mean daily high temperature (TempH) was 21.34 °C. The mean daily concentrations of PM2.5, PM10, SO2, NO2, CO, and O3-8h were 24.30 μg/m^3^, 36.25 μg/m^3^, 14.18 μg/m^3^, 0.54 μg/m^3^, 7.64 μg/m^3^, and 80.58 μg/m^3^, respectively. Detailed descriptive statistics are provided in Supplementary material 1. Figure 1 shows the daily time series of relative humidity, solar shortwave radiation, daily minimum temperature, and daily maximum temperature in Zunyi from 2023 to 2025. Temperature and SWR exhibited clear periodic patterns. Spearman correlation analysis was used to assess correlations between air pollutants and meteorological variables. Potential confounders significantly correlated with the meteorological exposure of interest were adjusted for in the corresponding model to reduce potential analytical bias. The correlation results are provided in Supplementary material 1.

### 3.2 Overall associations of meteorological factors with BPV

**Figure 2:**
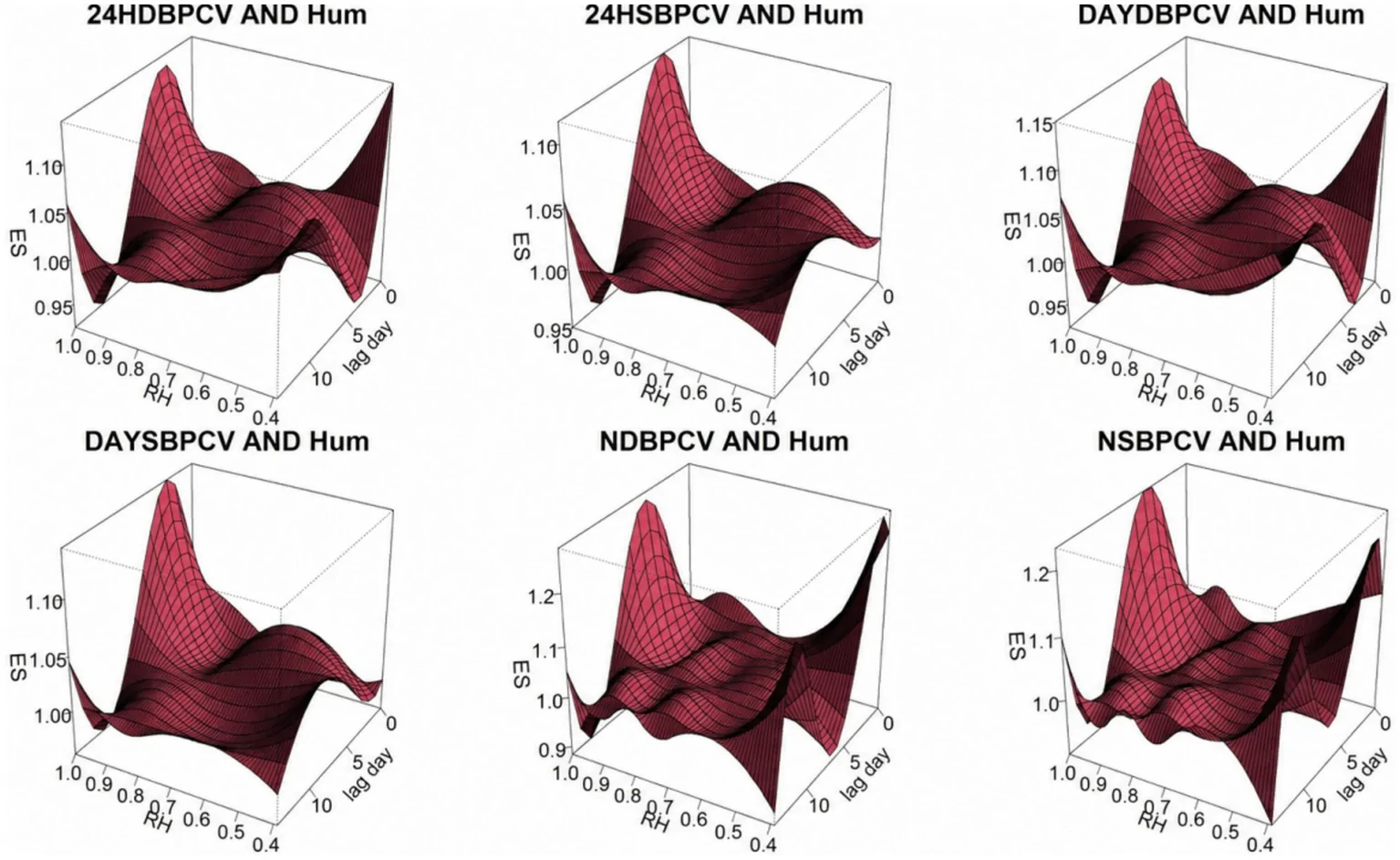
Three-dimensional plot of the cumulative exposure-lag-response association between relative humidity and BPV coefficients.

Across the 14-day observation window, Hum, SWR, TempL, and TempH showed non-linear and cumulative lagged associations with BPV among patients with primary hypertension. Higher Hum was positively associated with BPV coefficients across all monitoring periods, with the association peaking at approximately lag day 5; the association appeared more pronounced for systolic BPV. Lower SWR was positively associated with greater BPV at lag days 3–6 and appeared to be more strongly associated with daytime BPV. The overall cumulative lag patterns are shown in Figure 2 and Supplementary material 1.

### 3.3 Cumulative lagged associations of meteorological factors with BPV

**Figure 3:**
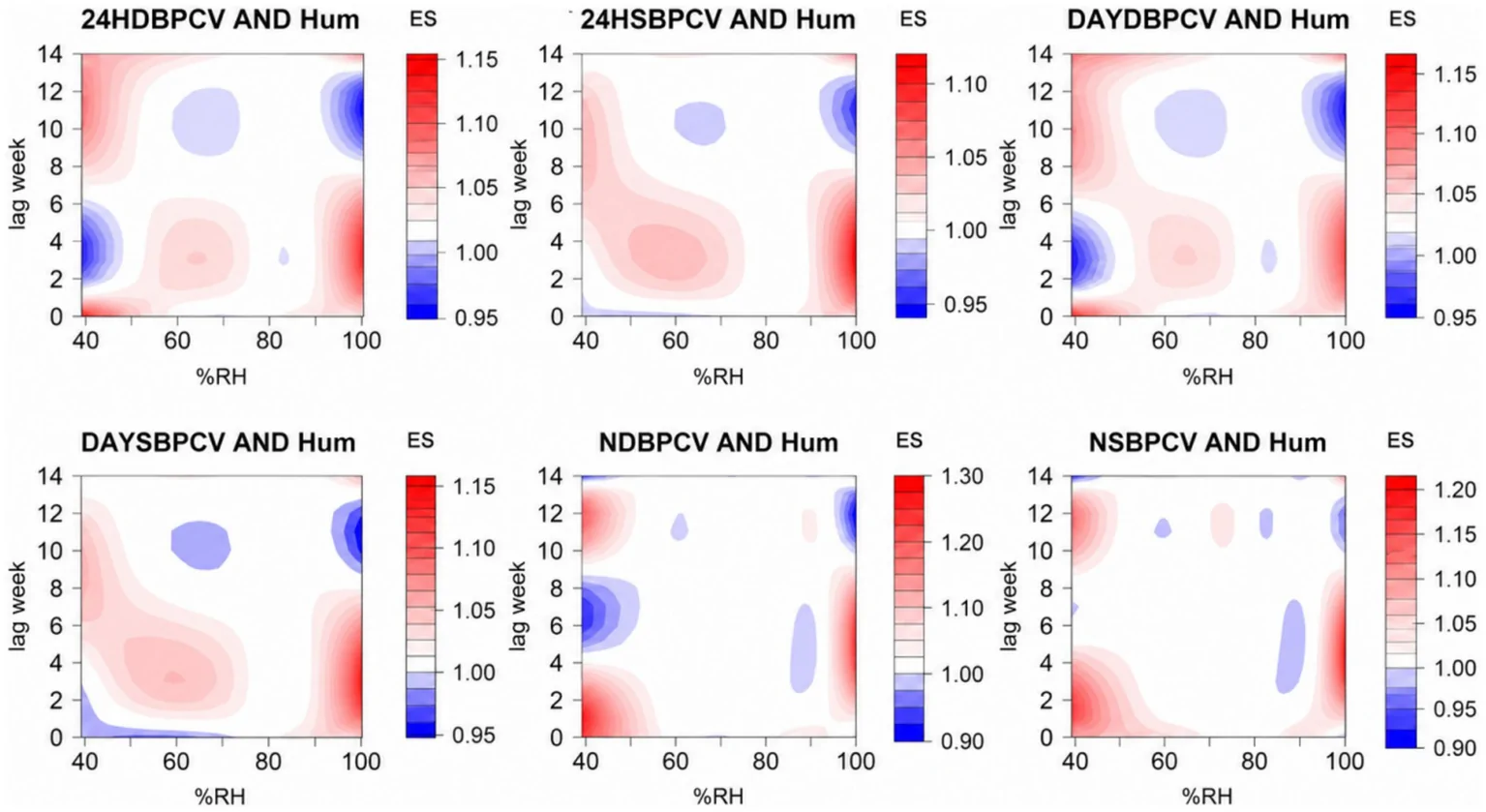
Contour plots of the cumulative exposure-lag-response association between relative humidity and BPV indices.

Low Hum (P1, 50.07%) was not significantly associated with BPV. In contrast, high Hum (P99, 97.07%) showed non-linear positive associations with 24HDBPCV, 24HSBPCV, DAYDBPCV, DAYSBPCV, NDBPCV, and NSBPCV over lag windows of 2–10, 3–10, 3–12, 3–9, 4–11, and 5–11 days, respectively. At lag 7 days, the corresponding effect estimates (95% CIs) were 1.53 (1.17–1.99), 1.56 (1.17–2.08), 1.65 (1.25–2.19), 1.54 (1.13–2.09), 1.75 (1.19–2.56), and 1.79 (1.21–2.65), respectively (Figure 3 and Table 3).

**Table 3:** Cumulative lagged associations of the main meteorological exposures with BPV indices.

Exposure contrast	BPV index	Main lag window	ES (95% CI)
High Hum (P99)	24HDBPCV	2–10 days	1.53(1.17,1.99)
High Hum (P99)	24HSBPCV	3–10 days	1.56(1.17,2.08)
High Hum (P99)	DAYDBPCV	3–12 days	1.65(1.25,2.19)
High Hum (P99)	DAYSBPCV	3–9 days	1.54(1.13,2.09)
High Hum (P99)	NDBPCV	4–11 days	1.75(1.19,2.56)
High Hum (P99)	NSBPCV	5–11 days	1.79(1.21,2.65)
Low SWR (P1)	24HDBPCV	6–10 days	1.36(1.03,1.80)
Low SWR (P1)	DAYDBPCV	5–10 days	1.39(1.04,1.87)
Low SWR (P1)	DAYSBPCV	3–9 days	1.35(1.03,1.76)

High SWR (P99, 25.86 MJ/m^2^) was not significantly associated with BPV. Low SWR (P1, 1.27 MJ/m^2^) showed non-linear positive associations with 24HDBPCV, DAYDBPCV, and DAYSBPCV over lag windows of 6–10, 5–10, and 3–9 days, respectively. At lag 7 days, the corresponding effect estimates (95% CIs) were 1.36 (1.03–1.80), 1.39 (1.04–1.87), and 1.35 (1.03–1.76), respectively. The relevant plots are provided in Supplementary material 1, and detailed effect estimates are reported in Supplementary material 2.

### 3.4 Lag-specific associations of meteorological factors with BPV

**Figure 4:**
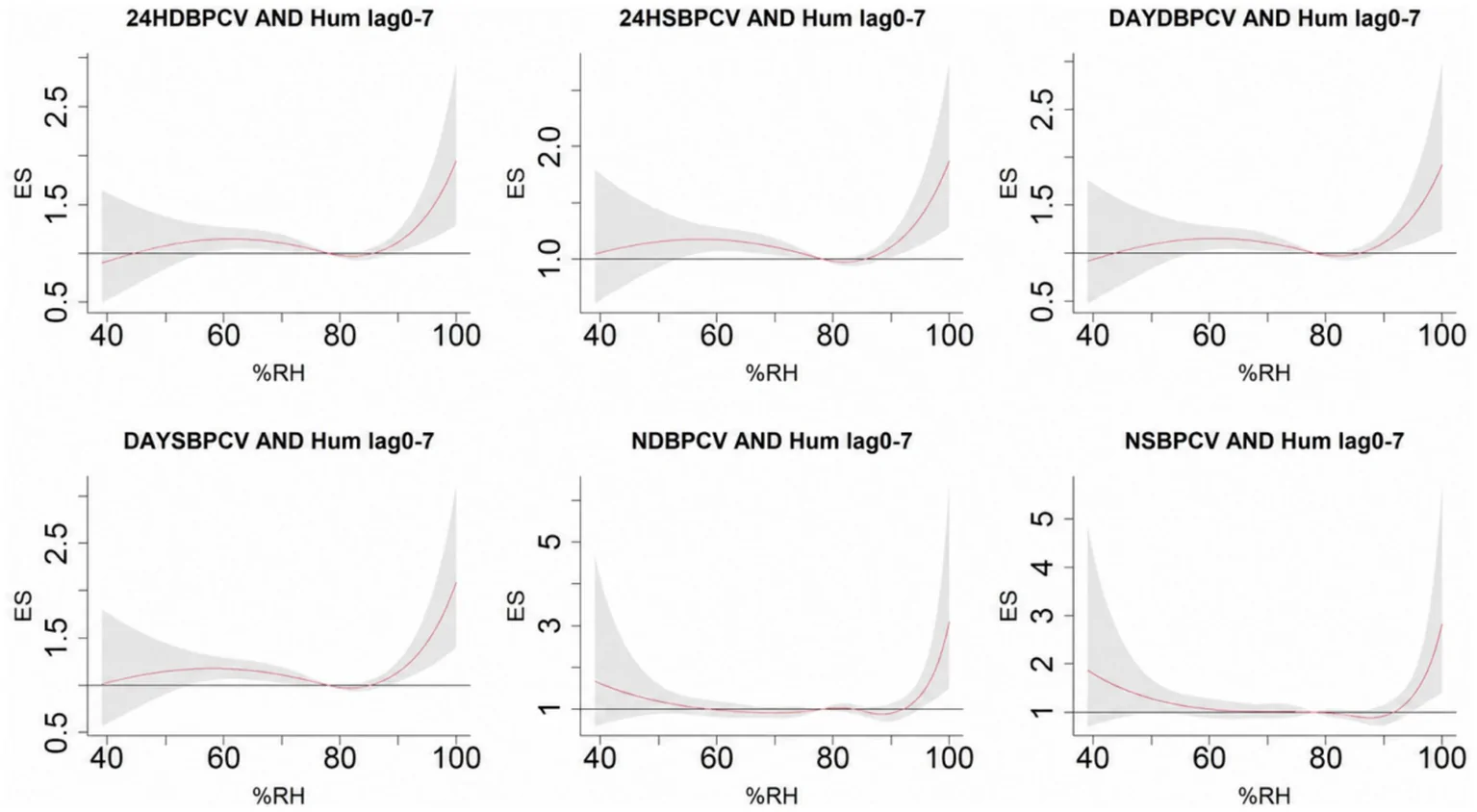
Lag-specific effect estimates for high relative humidity and BPV indices over the 14-day observation period.

Lag-specific analyses showed that high relative humidity (P99, 97.07%) was positively associated with changes in 24HDBPCV, 24HSBPCV, DAYDBPCV, DAYSBPCV, NDBPCV, and NSBPCV at lag days 2–6, 1–6, 2–6, 1–6, 3–8, and 2–6, respectively (Figure 4). Low SWR was positively associated with changes in 24HDBPCV, DAYDBPCV, and DAYSBPCV at lag days 4–6, 4–6, and 4, respectively. Lag-specific effect estimates are presented in Supplementary materials 1, 2.

### 3.5 Exposure-response associations between meteorological factors and BPV

**Figure 5:**
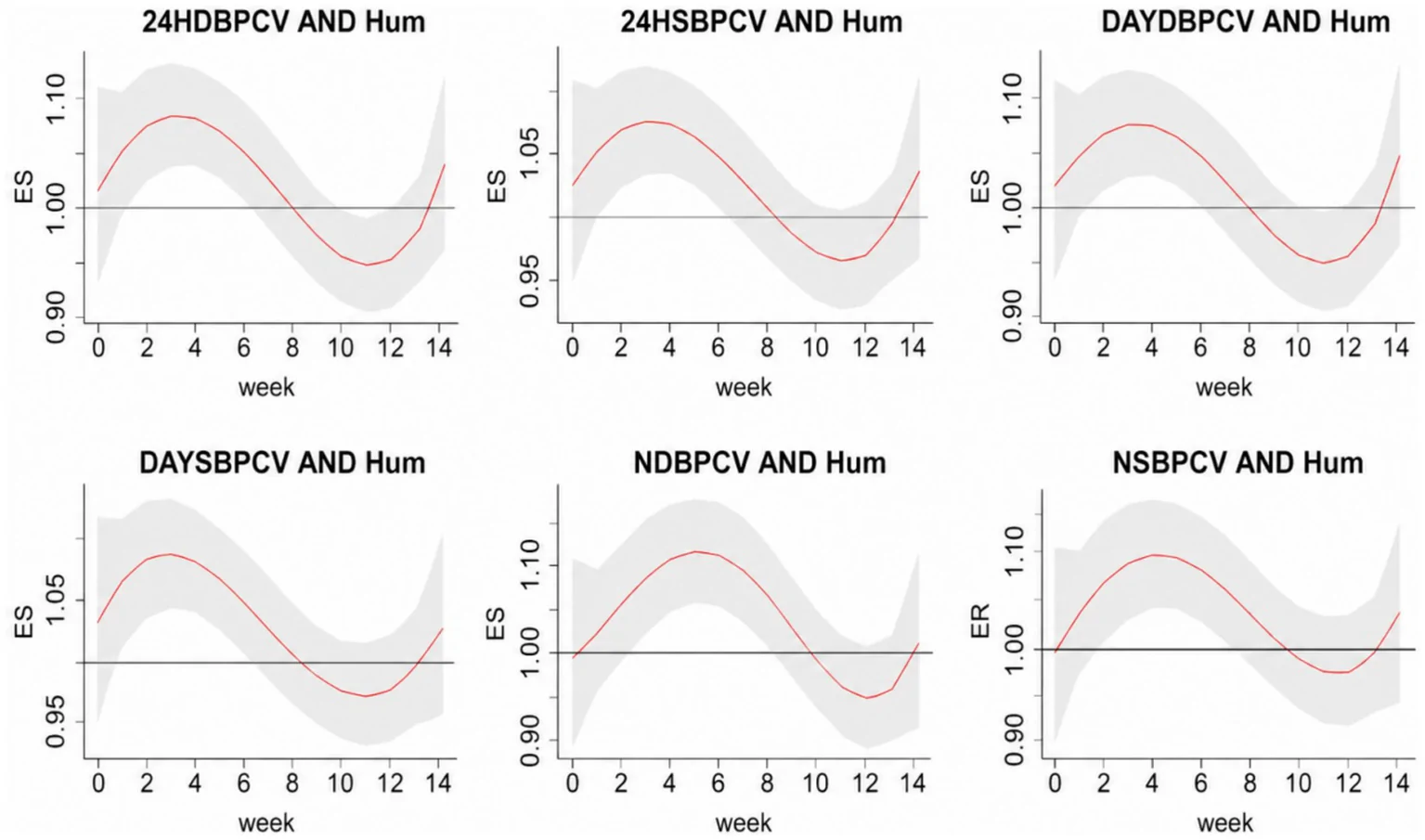
Time–exposure–response curves for relative humidity and blood pressure variability indices.

In the exposure-response analyses, in addition to the associations observed at high Hum (P99, 97.07%), moderate Hum (60–65%) was positively associated with changes in 24HDBPCV, 24HSBPCV, DAYDBPCV, and DAYSBPCV (Figures 5A–D). Low SWR (1–3 MJ/m^2^) was also positively associated with changes in 24HDBPCV, 24HSBPCV, DAYDBPCV, and DAYSBPCV. Detailed effect estimates are provided in Supplementary materials 1, 2.

### 3.6 Subgroup analyses of the cumulative associations of meteorological factors with BPV

**Figure 6:**
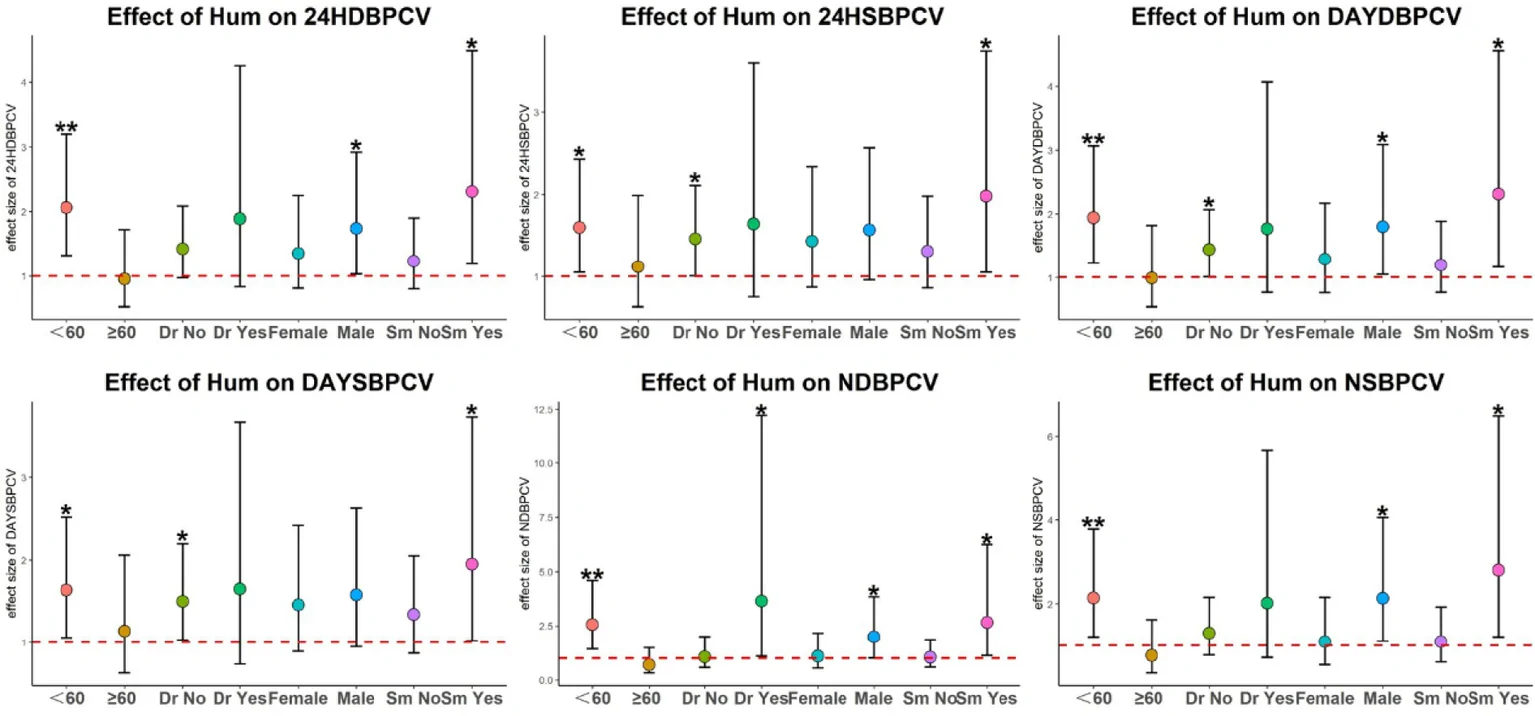
Subgroup-specific associations between relative humidity and BPV. Dr. No, no alcohol consumption; Dr. Yes, alcohol consumption; Sm No, nonsmoker; Sm Yes, smoker.

Z tests were used to evaluate statistical differences between subgroups; detailed results are provided in Supplementary material 3. In analyses of the 7-day cumulative lagged association stratified by age, sex, smoking status, and alcohol consumption, high Hum (97.07%) was positively associated with increased BPV coefficients in the daily series for participants aged <60 years, men, and smokers (Figure 6).

In stratified analyses of the 7-day cumulative lagged association for SWR, low SWR (1.27 MJ/m^2^) was positively associated with higher 24HDBPCV, DAYDBPCV, and DAYSBPCV in the daily series for men, with effect estimates (95% CIs) of 1.69 (1.04–2.74), 1.78 (1.08–2.94), and 1.67 (1.05–2.68), respectively. No significant between-subgroup differences were observed for age, smoking status, or alcohol consumption. Because these comparisons were based on daily aggregated series, they cannot establish greater individual susceptibility within the corresponding patient categories. The subgroup findings should therefore be considered exploratory. Complete subgroup results, including Z-test *p* values and q values, should be reported in Supplementary material 3.

### 3.7 Changes in BPV across meteorological exposure levels

**Figure 7:**
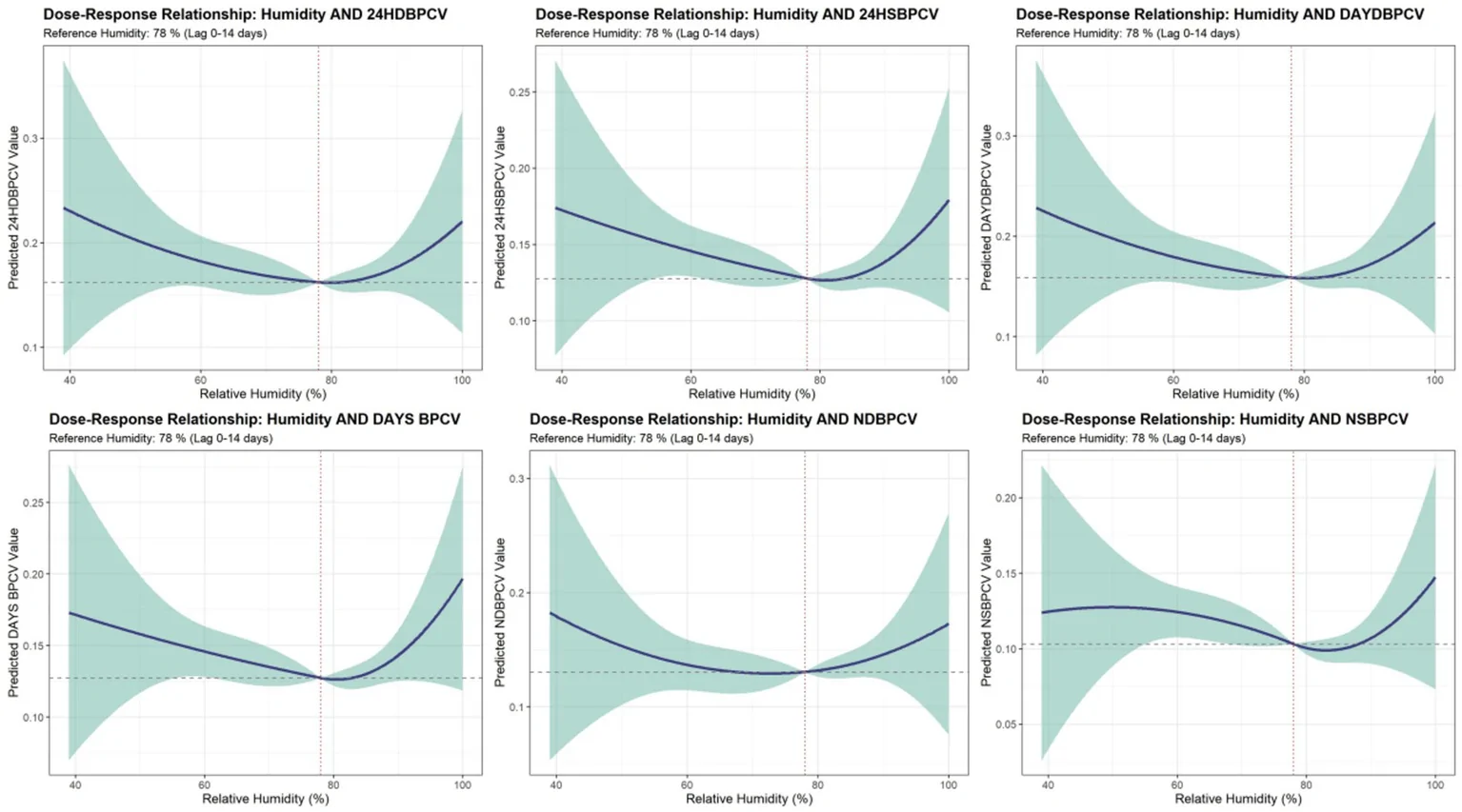
Changes in BPV indices across levels of relative humidity.

The relationships between meteorological exposure levels and BPV are shown in Figure 7 and Supplementary material 4. Over the 14-day exposure window, beginning at the P55 level of relative humidity (80%), each 5-percentage-point increase in relative humidity was associated with mean changes of 0.48% (95% CI, −0.29 to 1.25%), 0.42% (−0.11 to 0.95%), 0.45% (−0.35 to 1.25%), 0.57% (0.04 to 1.13%), 0.35% (−0.35 to 1.06%), and 0.35% (−0.18 to 0.89%) in 24HDBPCV, 24HSBPCV, DAYDBPCV, DAYSBPCV, NDBPCV, and NSBPCV, respectively.

**Figure 8:**
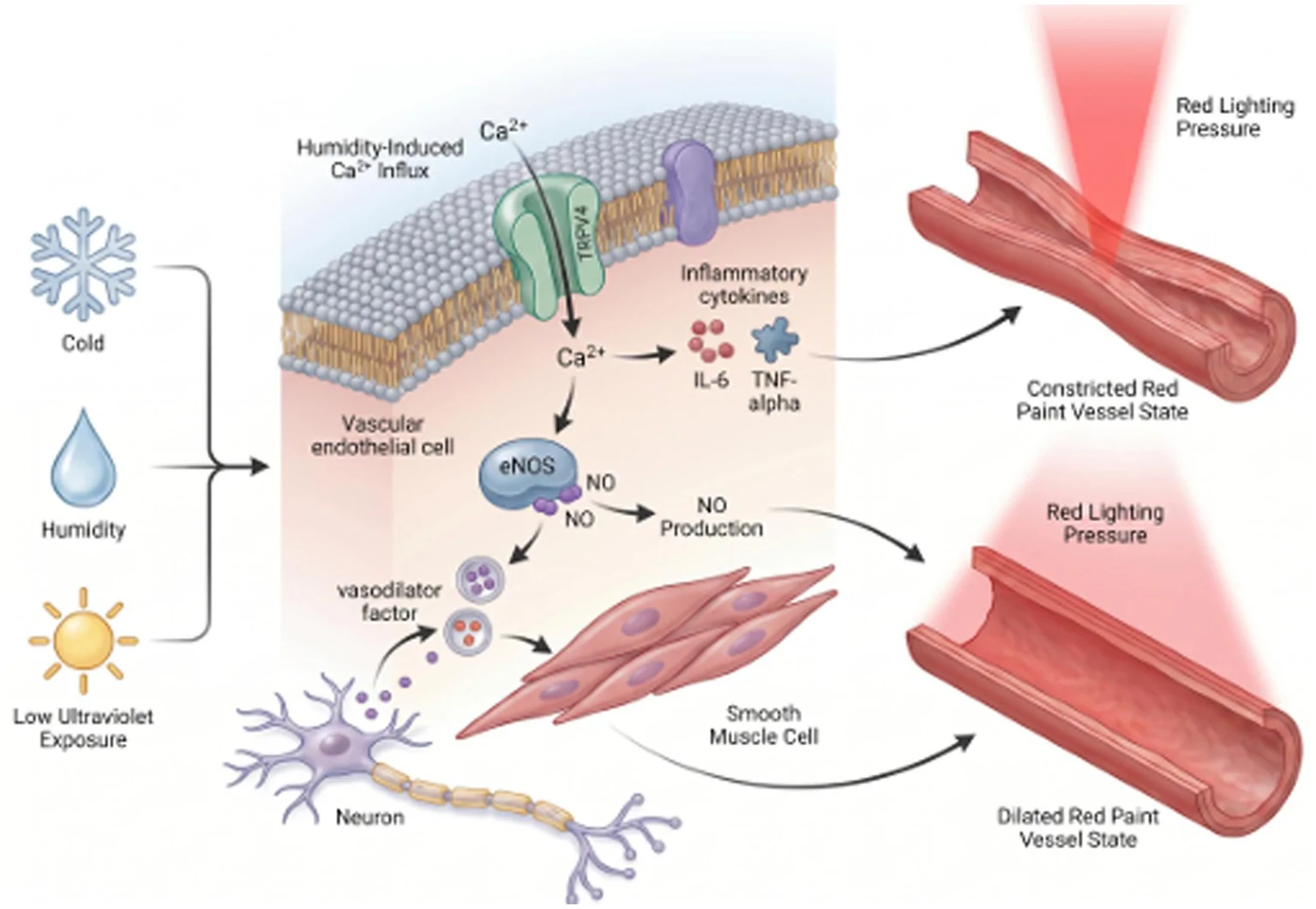
The potential mechanism diagram of meteorological factors mediating vasoconstriction and vasodilation. Temperature, relative humidity, and shortwave radiation may affect vascular smooth muscle contraction and relaxation by modulating calcium influx into vascular endothelial cells, inflammatory cytokine release, and the eNOS/NO signaling pathway, thereby contributing to blood pressure fluctuations.

Across SWR levels from P1 (1.3 MJ/m^2^) to P45 (9.6 MJ/m^2^), each 5-percentile increase in SWR was associated with mean decreases of 0.64% (95% CI, −0.01 to 1.28%), 0.29% (−0.15 to 0.73%), 0.66% (0.01 to 1.33%), and 0.50% (0.03 to 0.97%) in 24HDBPCV, 24HSBPCV, DAYDBPCV, and DAYSBPCV, respectively (Tables 3, 4).

**Table 4:** Summary of the main subgroup findings.

Exposure	Subgroup	BPV index	ES (95% CI)
High Hum (P99)	Age <60 years	24HDBPCV	2.06(1.32,3.20)
High Hum (P99)	Men	24HDBPCV	1.74(1.03,2.92)
High Hum (P99)	Smokers	24HDBPCV	2.31(1.19,4.50)
High Hum (P99)	Alcohol consumers	NDBPCV	3.65(1.09,12.22)
Low SWR (P1)	Men	DAYDBPCV	1.78(1.08,2.94)

### 3.8 Sensitivity analysis

Sensitivity analyses were conducted by increasing the df for meteorological factors from 3 to 5 and the df for the temporal trend from 7 to 9. Although the locations of some effect peaks shifted slightly, the overall patterns remained materially unchanged, indicating that the estimated exposure-response associations between meteorological factors and BPV were robust. Detailed results are provided in Supplementary material 1.

## 4 Discussion

Using DLNMs and accounting for the regional meteorological characteristics of Zunyi, this study evaluated the short-term associations between meteorological factors and BPV. Higher relative humidity and lower shortwave radiation showed non-linear associations with greater BPV. These associations were not immediate but exhibited distinct lag structures. A longitudinal study conducted in China similarly reported that lower outdoor temperature was significantly associated with increased BPV and blood pressure load after a lag of approximately 20 h (38). That study supports the biological and temporal plausibility of lagged associations between meteorological exposures and blood pressure indices; however, it had a different temporal resolution and focused primarily on temperature. The present study extends previous work by evaluating relative humidity and shortwave radiation and by characterizing non-linear cumulative associations over a maximum lag of 14 days.

Substantial evidence indicates that abrupt blood pressure fluctuations increase the risk of adverse cardiovascular events and that meteorological factors are closely associated with both blood pressure levels and BPV (39, 40). A large study of approximately 450,000 individuals in China reported U-shaped associations between temperature and blood pressure in temperate monsoon and continental climatic regions. Each 10 °C decrease in temperature was associated with mean increases of 0.74 mmHg in systolic blood pressure and 0.60 mmHg in diastolic blood pressure, with individuals in subtropical regions showing greater sensitivity to low temperatures (41). Another study suggested that 20–25 °C may represent a relatively favorable temperature range for stable blood pressure (42). The effects of temperature on blood pressure and BPV may involve endothelial dysfunction and activation of the sympathetic nervous system and RAAS. Cold exposure may reduce vascular endothelial compliance and decrease endothelial nitric oxide synthase expression, thereby increasing blood pressure (43). It may also activate sympathetic outflow and impair arterial baroreflex function (44); both baroreflex dysfunction and enhanced sympathetic activity can further increase BPV (45). With respect to ultraviolet radiation, a component of shortwave radiation, Veleva et al. found an inverse association with hypertension, although the blood pressure-lowering effect was short term and no sustained effect was observed (46). Weller et al. similarly reported an association between incident solar ultraviolet radiation and lower systolic blood pressure in a US population (47). Potential mechanisms include ultraviolet-induced release of endothelial nitric oxide and modulation of vitamin D synthesis, which may promote vasodilation and thereby influence blood pressure and its variability (48, 49). A Chinese study also reported a U-shaped association between relative humidity and blood pressure, with greater sensitivity to humidity changes among patients aged <60 years, women, and those not receiving antihypertensive medication (9).

In the present study, both high (>90%) and moderate (60–70%) relative humidity were positively associated with greater BPV. Beginning at the P55 level of relative humidity (80%), each 5-percentage-point increase was associated with mean increases of 0.48, 0.42, 0.45, 0.57, 0.35, and 0.35% in 24HDBPCV, 24HSBPCV, DAYDBPCV, DAYSBPCV, NDBPCV, and NSBPCV, respectively. These findings are broadly consistent with previous evidence. Guo et al. (50) showed that reducing indoor relative humidity significantly lowered levels of coagulation-related biomarkers (sCD40L and PAI-1) and inflammatory markers (CRP, IL-6, and TNF-*α*), reduced blood pressure and heart rate among older adults, and potentially decreased the risk of adverse cardiovascular events. Their analysis suggested that an indoor relative humidity of 46.9% ± 8.7% was most favorable for cardiovascular health. Potential mechanisms may involve vascular dysfunction, neurohumoral regulation, and psychological pathways. Experimental evidence has also shown that relative humidity of 90% may interact with fine particulate matter to activate the TRPV4 pathway, increase vasoconstrictor mediators, decrease vasodilator mediators, and thereby increase blood pressure and BPV (51). In addition, both excessively high and excessively low humidity may disrupt normal sleep architecture, and sleep disturbance may indirectly increase BPV (52).

Low shortwave radiation (1–3 MJ/m^2^) was positively associated with greater BPV in this study. Across SWR levels from P1 (1.3 MJ/m^2^) to P45 (9.6 MJ/m^2^), each 5-percentile increase was associated with mean decreases of 0.64, 0.29, 0.66, and 0.50% in 24HDBPCV, 24HSBPCV, DAYDBPCV, and DAYSBPCV, respectively. Biologically, sunlight may induce photolytic release of nitric oxide stored in the skin; additional mechanisms may involve vitamin D synthesis and modulation of sympathetic and baroreflex activity. These effects are generally short term and nonpersistent (53). A large US population study likewise found an inverse association between the ultraviolet component of shortwave radiation and systolic blood pressure (47). Low shortwave radiation may also alter pineal melatonin secretion and disrupt circadian rhythms, leading to sympathetic activation, vagal withdrawal, reduced heart rate variability, abnormal circadian blood pressure patterns, and attenuated nocturnal blood pressure dipping (a nondipping pattern) (54).

Regarding temperature, a study of older Chinese adults found a significant inverse association between morning indoor temperature below 18 °C and systolic blood pressure. Morning temperature fluctuations also had an independent effect on BPV, with fluctuations exceeding 1.1 °C significantly increasing BPV (26). Individuals who have lived in cold regions for long periods may exhibit greater adaptation to low temperatures and a weaker stress response. Previous studies have linked increased BPV to enhanced sympathetic-mediated vascular reactivity to environmental stress (55). Definitions of extremely low temperature vary across studies because the absolute exposure temperature is region specific (56). Consequently, abrupt regional temperature declines may transiently increase BPV. Xu et al. (57) also reported that decreases in ambient temperature were associated with acute increases in blood pressure and BPV; these associations attenuated over time and disappeared after approximately 10 h. Compared with a thermoneutral environment (25 °C), exposure to a hot environment (35 °C) has been associated with impaired autonomic regulation and stronger cardiovascular responses, resulting in increased systolic BPV (58).

This study incorporated the distinctive meteorological characteristics of Zunyi and provides evidence regarding the associations between meteorological factors and BPV among patients with hypertension. Nevertheless, several limitations should be acknowledged. First, this was a single-center, retrospective study, which may limit the generalizability of the findings. The study can identify associations between meteorological factors and daily aggregated BPV among patients with hypertension but cannot provide direct evidence of individual susceptibility. In addition, exposure assessment was subject to measurement error because all environmental exposure data were obtained from fixed monitoring stations rather than from real-time individual-level measurements. Actual personal exposure may differ substantially from measurements recorded at monitoring stations, and this exposure misclassification may have affected the accuracy of the estimates. Second, although common hypertension-related risk factors were statistically adjusted for to control known confounding, residual confounding remains possible because of the observational design. Potential confounders not included in the analysis, such as individual antihypertensive medication use and long-term dietary patterns, may have influenced the robustness of the results. Finally, this study identified associations between meteorological factors and BPV but did not directly investigate the underlying pathophysiological mechanisms or evaluate the relationship between BPV and subsequent clinical outcomes.

Future studies should address these limitations to clarify the mechanisms through which meteorological factors influence BPV. Priorities include multicenter prospective cohort studies with rigorous control of potential confounding and further optimization of analytical models; incorporation of personal microenvironmental monitoring devices to improve individual-level exposure assessment; and randomized controlled intervention studies to evaluate personalized blood pressure management strategies based on meteorological early warnings. A region-specific meteorological risk prediction model for hypertension in Zunyi and a mobile application-based warning system could also be developed to improve clinical applicability. These methodological advances would strengthen the reliability and translational value of the findings.

## 5 Conclusion

Using a DLNM framework and accounting for the meteorological characteristics of Zunyi, this study found that higher relative humidity and lower shortwave radiation were associated with increased BPV over cumulative lag periods. The magnitude of these associations may differ across aggregated daily series defined by demographic and behavioral characteristics. These findings require confirmation in multicenter studies with individual-level exposure assessment.

## Data Availability

The original contributions presented in the study are included in the article/Supplementary material, further inquiries can be directed to the corresponding author/s.
