# Supplementary material for: Association between meteorological factors and blood pressure variability in northern Guizhou, China
Source: Front Public Health. 2026 Aug 7;14:1888902. doi: 10.3389/fpubh.2026.1888902 (PMC13493591; doi:10.3389/fpubh.2026.1888902)
Supplement: Supplementary file 1 [file Table_1.docx]

Table 1 Meteorological Factors and Air Pollution Levels

| **Variable** | **Maximum** | **Minimum** | **Mean** |
| --- | --- | --- | --- |
| **Meteorological factors** |  |  |  |
| Hum(%) | 100 | 39 | 77.14 |
| SWR(MJ/m²) | 28.35 | 0.96 | 11.46 |
| TempL(℃) | 25.95 | -4.2 | 14.02 |
| TempH(℃) | 37.5 | -2.1 | 21.34 |
| **Air pollution** |  |  |  |
| PM2.5(µg/m^3^） | 114 | 5 | 24.3 |
| PM10(µg/m^3^） | 315 | 8 | 36.25 |
| NO_2_(µg/m^3^） | 43 | 5 | 14.18 |
| CO(µg/m^3^） | 1.1 | 0.2 | 0.54 |
| SO_2_(µg/m^3^） | 64 | 3 | 7.64 |
| O_3__8h(µg/m^3^） | 174 | 0 | 80.58 |

Figure 1: Correlation analysis between air pollutants and meteorological factors

Figure 2: 3D surface plot of the time-dose cumulative effect of solar shortwave radiation on four coefficients of variation of blood pressure

Figure 3: 3D surface plot of the time-dose cumulative effect of daily minimum temperature on the coefficient of variation of blood pressure

Figure 4: 3D surface plot of the time-dose cumulative effect of daily maximum temperature on the coefficient of variation of blood pressure

Figure 5: Contour plot of the time-dose cumulative effect of SWR on blood pressure variability related indicators

Figure 6: Contour plot of the time-dose cumulative effect of TempL on blood pressure variability related indicators

Figure 7: Contour plot of the time-dose cumulative effect of TempH on blood pressure variability related indicators

Figure 8: Daily exposure risk effect plot of low SWR on blood pressure variability related indicators during the 14-day observation period

Figure 9: Daily exposure risk effect plot of TempH on blood pressure variability related indicators during the 14-day observation period

Figure 10: Dose cumulative effect plot of SWR on blood pressure variability related indicators with a 7-day lag

Figure 11: Dose cumulative effect plot of TempL on blood pressure variability related indicators with a 7-day lag

Figure 12: Dose cumulative effect plot of TempH on blood pressure variability related indicators with a 7-day lag

Figure 13: Effect value plot of blood pressure variability related indicators in each subgroup under exposure to low SWR (1.27 MJ/m²)

Figure 14: Effect value plot of blood pressure variability related indicators in each subgroup under exposure to extreme high temperature (35.57 ℃) and extreme low temperature (-1.82 ℃)

Figure 15: Trends of blood pressure variability indicators across different shortwave radiation levels

Figure 16: Trends of blood pressure variability indicators across different TempL levels

Figure 17: Trends of blood pressure variability indicators across different TempH levels

Figure 18: Sensitivity analysis of the correlation between each coefficient of variation of blood pressure and Hum

Figure 19: Sensitivity analysis of the correlation between each coefficient of variation of blood pressure and SWR

Figure 20: Sensitivity analysis of the correlation between each coefficient of variation of blood pressure and TempL

Figure 21: Sensitivity analysis of the correlation between each coefficient of variation of blood pressure and TempH
